# A Novel Tool for Classification of Epidemiological Data of Vector-Borne Diseases

**DOI:** 10.4103/0974-777X.59248

**Published:** 2010

**Authors:** Sree Hari Rao Vadrevu, Suryanarayana U Murty

**Affiliations:** *Department of Mathematics and Statistics 202, Rolla Building 400 W 12^th^ Street ROLLA, USA*; 1 *Biology Division, Indian Institute of Chemical Technology, Hyderabad - 500 018, India*

**Keywords:** Epidemiology, Vector-borne disease, Filariasis, *k*-nearest neighbor algorithm, VBClassif

## Abstract

In this article we present a novel tool that renders efficient classification of epidemiological data of vector-borne diseases. This algorithm has been applied on the data of the Filariasis disease and the results are compared with the well-known *k*-nearest neighbor algorithm.

## INTRODUCTION

‘Crude classification and false generalizations are the curse of organized life.’ However, that does not halt us from classifying objects and extending these generalizations to a vast category. Scientists have been classifying objects since time immemorial and this effort to group the objects so that they fit nicely together with other similar objects has led to the simplification and organization of an otherwise chaotic world. Classification in biology dates back to Aristotle's time and has now reached such sophistication that human effort is often minimized and its place has been superseded by state -of-the-art computer-aided tools. With the accelerating advances in high throughput methods, the generation of data is increasing exponentially. An astonishingly high rate of data generation and flow of data has not kept pace with the development of accurate and precise classification tools that can bridge the gap between demand and supply. In the biological field, classification tools are used vigorously in taxonomical classification to emulate the ambiguities in assigning the appropriate rank.[1,2] Several applications can be found with classification tools, such as, gene expression analysis,[3,4] structural classification of proteins (SCOP),[5] classification of methylation array data, and protein proteomic analysis for cancer,[6,7] for elucidating the evolutionary distance among several species, comparison of biological sequences,[8] protein structure prediction,[9] assigning scores to drug molecules in docking studies, and in ranking the small molecules based on their structure activity relationship (QSAR)[10] and structure property relationship (QSPR). These classification tools are mainly based on hardcore statistics and mathematical models. Principal component analysis (PCA), partial least square method, and the regression analysis method lie in the heart of these tools. Among the myriad of classification tools, HCA (Hierarchical Cluster Analysis), MDS (Multi Dimensional Scaling), CART (Classification and Regression Tree), FP (Frequency Pattern) Tree, SOM (Self Organizing Maps), Correlation coefficient clustering, SVM (Support Vector Machine), BP-ANN (Back Propagation Artificial Neural Network), GA (Genetic Algorithm), Genetic Programming, and k-Means and k-nearest neighbor (kNN) are some of them. Vector-borne diseases often tend to be complicated and the enormous data generating from the experimental studies warrant a need for efficient tools, for analysis. Although computer technology is undergoing a revolution its application in classification is still in its infancy, its potential, especially, has not been tapped in the arena of vector-borne diseases. Often, the magnitude of epidemiological data and its vast array of types poses a challenge for an epidemiologist. In our gold rush to information, we end up settling for a compromise between accuracy and speed. One of the common statistical tools used in these kinds of studies is the kNN.

The present article is organized as follows: In Section 2, we describe the kNN approach and in Section 3 we present the methodology, the data sets, and data normalization. The main algorithm is discussed in Section 4. The results and performance evaluation of the novel algorithm form the content of Section 5, while the conclusions are deferred to Section 6.

### Nearest neighbor

The kNN algorithm is a simple instance-based learning method for performing general, nonparametric classification. First introduced by the researchers E. Fix and J. Hodges in their article, ‘Discriminatory Analysis: Nonparametric Discrimination: Consistency Properties’, in 1951, it is well explored in literature and has been seen to have a good classification (prediction) performance on a wide range of real world data sets, Xiong and Chen (2006). It is simple and straight forward to implement. kNN is based on a distance function for pairs of observations, such as the Euclidean distance. In this classification paradigm, the *k*-nearest neighbors of a training data are computed first. Then the similarities of one sample from the testing data to the *k*-nearest neighbors are aggregated according to the class of the neighbors, and the testing sample is assigned to the most similar class. The performance of the kNN algorithm is influenced by the following three main factors:

1. The distance metric used to locate the nearest neighbors
2. The decision rule used to derive a classification from the *k*-nearest neighbors
3. The number of neighbors used to classify the new sample

kNN classifiers are well-suited to solve the given problem because they do not have to spend additional effort on distinguishing additional classes. One advantage of kNN is that it is well-suited for multi-modal classes as its classification decision is based on a small neighborhood of similar objects. Therefore, even if the target class is multi-modal (i.e., consists of objects whose independent variables have different characteristics for different subsets), it can still lead to good accuracy.

Some pleasant aspects of the nearest neighbor (NN) classifier:

1. Many other techniques (such as, decision trees and linear discriminants) require the explicit construction of a feature space, which for some distance functions is intractable
2. The NN classifier effortlessly deals with the hugely multiclass nature of visual object recognition
3. From a theoretical point of view, it has the remarkable property wherein, under very mild conditions, the error rate of a kNN classifier tends toward the Bayes optimal as the sample size tends toward infinity
4. Additionally, kNN classifiers can be applied to any type of object representation as long as a distance measure is available

Unfortunately, kNN classification has a major drawback as well. The efficiency of classification rapidly decreases with the number of training objects.

*k*-nearest neighbor classifier: A major drawback of the similarity measure used in kNN is that it uses all features equally in computing similarities. This can lead to poor similarity measures and classification errors, when only a small subset of the features is useful for classification. The accuracy of the kNN algorithm can be severely degraded by the presence of noisy or irrelevant features, or if the feature scales are not consistent with their relevance. The main weakness of this technique is that performance is subject to the often *ad hoc* choice of the similarity metric, especially for heterogeneous datasets, from which the derived features are of different types and scales, and are correlated. In addition, the standard kNN methods suffer from the curse of dimensionality. The neighborhood of a given point becomes very sparse in a high dimensional space, resulting in high variance. The algorithm is easy to implement, but it is computationally intensive, especially when the size of the training set grows. There can be no tie. These resulting class labels are used to classify each data point in the data set. However, due to the shortcomings faced by kNN, an imperative need is felt for a more robust tool with high efficiency and accuracy.

**Figure 1:**
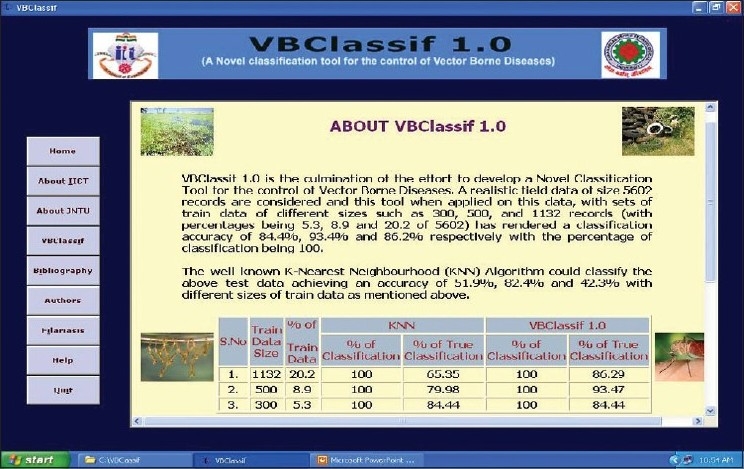
Snapshot showing VBClassif 1.0 application window

On the basis of the kNN approach, a novel tool VBClassif ver.1.0, for classification of epidemiological data of vector-borne diseases, was developed [Figure 1].

VBClassif is a novel efficient classification algorithm (designated as VBClassif 1.0), which classifies the records of those affected by Filariasis. This software has been utilized to classify up to a 1,00,000 records and the effective classification yield percentage is 94%.

## METHODOLOGY

The data on various parameters like age, sex, class, and so on, of different individuals affected by Filariasis is considered. The program is trained with a small portion of data designated as train data and is then tested for efficiency by executing on the test data, which is expectedly large. The algorithm is tested with minimal train data and observed to see if the efficiency increases or remains unaltered.

### Dataset

A real life dataset, amounting to a data size of 5602 records, pertaining to a major disabling vector-borne disease Filariasis was used for this study. Epidemiological, socioeconomic, and entomological data was collected from East and West Godavari districts of Andhra Pradesh and considered for this study.

### Data normalization

The data was normalized and each record was assigned a value of 0 or 1, based on the presence or absence of the disease characteristics.

Algorithm: The following is the main algorithm.

1. **Start**
2. **From the entire given test data**
  1. Separate ‘0’ class records and ‘1’ class records in the train data
  2. Take a record from the test data and calculate the Euclidean distance for each record in the train data for both the classes, separately
  3. Take least distance in ‘0’ class and ‘1’ class
  4. Compare the least distance in both the classes; put the query record in the class that is having the least distance
  5. If distances are equal those are taken as unclassified records
  6. Repeat steps 2 to 5 for every record in the test data.
3. **Now, on the unclassified records from the above-mentioned stage perform the following steps**
  7. Take the least distance record (say *r*_1_) in the ‘0’ class and least distance record (say *r*_2_) in the ‘1’ class, records that are classified.
  8. Take the record from the unclassified list that is having minimum distance (say *u*_1_).
  9. If *u*_1_ is less than both *r*_1_ and *r*_2_ then take the minimum of *r*_1_ and *r*_2_.
  10. If *u*_1_ is less then *r*_1_ than assign *u*_1_ to ‘0’ class. If *u*_1_ is less than *r*_2_ than assign *u*_1_ to ‘1’ class.
  11. Repeat steps 3 and 4 for all unclassified records from Step 2 of this method.
4. **Again, on the unclassified records from the above-**mentioned stage perform the following steps
  12. Calculate least *L*_1_, *L*_2_ and highest *H*_1_, *H*_2_ distances in ‘0’ and ‘1’ classes respectively.
  13. Calculate average ‘*r*’ as *r* = H1+L22.
  14. Calculate the distance (*d*_1_ and *d*_2_) between a record in unclassified list and the record that is having *H*_1_ distance and *L*_2_ distance.
  15. If *d*_1_ <*r* then assign query record to ‘0’ class otherwise assign query record to ‘1’.
  16. Repeat the steps from 1 to 4 for each record in the unclassified list and also for those in the true negative list.
5. **On the unclassified list perform the following operations**
  17. Calculate the distances between a record in the unclassified list and the remaining records in the same list.
  If *x*_1_, *x*_2_, *x*_3_,….., *x*_n_ are records in the unclassified list calculate the distances (*x*_1_, *x*_2_),(*x*_1_, *x*_3_),….(*x*_1_, *x*_n_).
  18. Take the least distance (*d*_1_) from these calculated distances.
  19. Take least distances (*r*_1_, *r*_2_) in the ‘0’ and ‘1’ classes respectively.
  20. Compare *d*_1_ with the minimum of *r*_1_ and *r*_2_ (say *m*_1_).
  21. Assign *d*_1_ to the class to which *m*_1_ belongs.
  22. Repeat steps 1 to 5 for every record in the unclassified list and also in the true negatives list.
6. **Again on the unclassified list perform the following steps**
  23. Calculate the Euclidean distance between the origin and the record in the unclassified list.
  If *x*_1_, *x*_2_, *x*_3_,….., *x*_n_ are fields in the record then
  $R_{1}=\sqrt{{\left(x_{1}\right)}^{2}+{\left(x_{2}\right)}^{2}+\ldots .{\left(x_{n}\right)}^{2}}$
  24. Divide every field value by *R*_1_
  x1R1,x2R1,x3R1,……xnR1.
  25. Repeat steps 1 and 2 for the new field values i.e.,
  x1R1,x2R1,x3R1,……xnR1.
  26. Calculate the Euclidean distance between a record with new field values in the unclassified list and every record in the classified records of ‘0’ and ‘1’ classes.
  27. Take least distance in the ‘0’ class and ‘1’ class.
  28. Compare the least distance in both classes and put the query record in the class that is having the least distance.
  29. Repeat the steps from 1 to 6 for every record in the unclassified list and the true negative list
7. **End of algorithm**

## RESULTS

The results obtained with different magnitudes of train data are shown in the Table 1:

**Table 1:** Test and train data records

Total records	Number of test data records	Number of train data records	% of train data	% of true classification
4470	3370	1100	25	83.38
4470	3870	600	13.5	92.77
4470	3970	500	12	91.30
4470	4170	300	6.7	98.40

The algorithm has performed effectively, compared to other well-known classification algorithms such as the K-nearest neighborhood (kNN) algorithm. The comparison results are tabulated in Table 2.

**Table 2:** Performance evaluation and comparison

Total records	Number of test data records	Number of train data records	% of train data	% of true classification (kNN)	% of true classification (VBClassif 1.0)
4470	3370	1100	25	81.72	83.38
4470	3870	600	13.5	87.26	92.77
4470	3970	500	12	92.42	91.30
4470	4170	300	6.7	96.82	98.40

The results in Table 2 help one to conclude that it is not necessary to always have a large data of records for training purpose and what is important is always the minimal set of useful data records. Clearly, a set of 300 train data records could capture the full information and render most effective classifications and predictions.

When applied to data sets with train data of different sizes, such as, 300, 500, 600, and 1100 records (with percentages being 6.7, 12, 13.5, and 25 of 4470, VBClassif ver 1.0 has rendered a classification accuracy of 98.4, 91.3, 92.77, and 83.38, respectively, with the percentage of classification being 100, while the well-known *k*-nearest neighborhood (kNN) algorithm could classify the above-mentioned test data achieving an accuracy of 96.82, 92.42, 87.26, and 81.72, with different sizes of train data as mentioned earlier.

The tool demonstrates a high percentage of classification accuracy as compared to the well-known kNN, which is a desired feature. VBClassif is a menu-driven and user friendly, robust tool, which even a novice can apply, to analyze a vast amount of data. This tool offers an advantage of being applicable to two-tier classification environments. This tool can be extrapolated to any vector-borne disease, and hence, provides an effective way of emulating the disease.

## CONCLUSION

Artificial intelligence has opened a new window of opportunity and definitely has a long way to go in the area of data analysis. The enormity of the magnitude of data and its complex nature often perplexes epidemiologists. This is especially true in the case of vector-borne diseases, where a timely and precise understanding of the disease, during the decision-making process, comes in handy for curbing the disease in the event of an outbreak or epidemic. Often the public health officials feel the need for a correct identification of true positive cases. A tool that classifies diseases according to the presence or absence of a disease will help in devising a clear strategy in mass drug administration programs and will help in the proper targeting of patients and also in the efficient use of resources. Available and well-known statistical tools tend to compromise on accuracy, speed, and efficiency. Interactive Classification tools supported by AI (Artificial Intelligence), such as VBClassif 1.0, will definitely pave the way for more efficient disease control and will also help epidemiologists find quick solutions to classification problems.

## References

[1] Choi Serene Hyun-Jin, Nieminen Timo A, Bahr Mark, Bahr Nan (2002). Improving Behaviour Classification Consistency: A Technique From Biological Taxonomy. In Australian Association for Research in Education Annual Conference.

[2] Graham M, Kennedy JB, Hand C (1999). The challenge of visualising multiple overlapping classification hierarchies.

[3] Wang D, Lv Y, Guo Z, Li X, Li Y, Zhu J (2006). Effects of replacing the unreliable cDNA microarray measurements on the disease classification based on gene expression profiles and functional modules. Bioinformatics.

[4] Xiong H, Chen XW (2006). Kernel-based distance metric learning for microarray data classification. BMC Bioinformatics.

[5] Murzin AG, Brenner SE, Hubbard T, Chothia C (1995). SCOP: A structural classification of proteins database for the investigation of sequences and structures. J Mol Biol.

[6] Model F, Adorján P, Olek A, Piepenbrock C (2001). Feature selection for DNA methylation based cancer classification. Bioinformatics.

[7] Rätsch G, Sonnenburg S, Schäfer C (2006). Learning interpretable SVMs for biological sequence classification. BMC Bioinformatics.

[8] Orengo CA, Michie AD, Jones S, Jones DT, Swindells MB, Thornton JM (1997). CATH—A hierarchic classification of protein domain structures. Structure.

[9] Svetnik V, Liaw A, Tong C, Culberson JC, Sheridan RP, Feuston BP (2003). Random forest: A classification and regression tool for compound classification and QSAR modeling. J Chem Inf Comput Sci.

[10] Mitchell TM (1997). Machine learning.

